# Association of Lamotrigine Plasma Concentrations With Efficacy and Toxicity in Patients With Epilepsy: A Retrospective Study

**DOI:** 10.1097/FTD.0000000000001205

**Published:** 2024-06-28

**Authors:** Ze-Ning Lee, Merel van Nuland, Tim Bognàr, Frans S. S. Leijten, Kim C. M. van der Elst

**Affiliations:** * Department of Clinical Pharmacy, University Medical Center Utrecht, Utrecht University, Utrecht, the Netherlands; and; † Department of Clinical Neurophysiology, Brain Center, University Medical Center Utrecht, Utrecht University, Utrecht, the Netherlands.

**Keywords:** epilepsy, lamotrigine, seizure freedom, toxicity, therapeutic drug monitoring

## Abstract

**Background:**

There is limited evidence to support the currently suggested lamotrigine (LTG) therapeutic reference range of 2.5–15 mg/L for the treatment of seizures. The objective of this study was to evaluate the association of LTG plasma concentrations with the efficacy and toxicity of the treatment in patients with epilepsy.

**Methods:**

Patients whose LTG plasma concentration was measured between January 2013 and February 2022 were included. Efficacy was defined as seizure freedom for at least 6 months around the time of measured LTG concentration. Toxicity was defined as any LTG-related adverse drug effect documented in each patient's health record or when the reason for measuring the LTG concentration was toxicity. In addition, the dose–concentration relationship of LTG was assessed.

**Results:**

In total, 549 concentrations from 259 patients with epilepsy were included. The most common reasons for therapeutic drug monitoring were suspected inefficacy (39%) and pregnancy (21%). The LTG plasma concentration was not associated with efficacy (adjusted odds ratio = 0.94; 95% confidence interval, 0.85–1.04). The LTG plasma concentration was positively associated with the incidence of toxicity after adjusting for age, sex, and number of antiepileptic drugs (odds ratio = 1.11; 95% confidence interval, 1.04–1.19). The daily dose had a significant linear correlation with the LTG plasma concentration (*P* < 0.001).

**Conclusions:**

The LTG plasma concentration was associated with toxicity, whereas no association with efficacy was found. A reference range of 2.5–10 mg/L may be considered to decrease the risk of toxicity while maintaining similar efficacy. Therapeutic drug monitoring may be useful when LTG-related toxicity is suspected and in cases of pharmacokinetic changes (eg, pregnancy and concomitant use of interacting drugs) that can influence the LTG plasma concentration.

## Abstract 2

Supplemental Digital Content is Available in the Text.

## INTRODUCTION

Epilepsy is a complex disorder that results in recurrent seizures, which burdens the life of many patients. Unfortunately, in 25%–30% of patients, optimal seizure control is not achieved with at least 2 antiepileptic drugs.^1^ For this reason, new antiepileptic drugs are increasingly being approved for use in clinical practice. Lamotrigine (LTG) is a newer generation antiepileptic drug. Studies have shown that 60%–70% of patients treated with LTG alone or in combination with valproic acid (VPA) achieve seizure freedom (SF) for at least 1 year.^2–4^ Although many patients benefit from this treatment, there is a significant variability in the clinical response. This is partly due to pharmacokinetic variability caused by genetic factors, age, clinical state, pregnancy, and concomitant use of interacting medications.^5–7^

Interestingly, an increase in both the incidence of adverse effects and seizure frequency is associated with supratherapeutic LTG plasma concentrations.^6–8^ Interpatient pharmacokinetic variability and a possible association of LTG concentrations with efficacy and toxicity indicate that LTG therapeutic drug monitoring (TDM) may be useful in optimizing seizure control while minimizing adverse effects in individual patients. Optimizing LTG exposure through TDM-guided dose adjustment may be valuable in cases of uncontrolled seizures, drug–drug interactions, comorbidities, suspected toxicity, nonadherence, and in specific patient populations such as children, the elderly, and women who are pregnant.^5^

Currently, the suggested LTG reference range for the treatment of seizures is 2.5–15 mg/L.^9^ However, there is limited evidence to support this range, and inconsistencies exist in the upper and lower limits reported in the literature. Most studies indicate that there is an overlap in plasma concentrations of responders and nonresponders, as well as in patients with and without adverse effects, and that further research is required to establish a well-defined therapeutic range for LTG.^6–12^ Gaining more insight into the optimal therapeutic exposure of LTG may help improve efficacy and minimize toxicity.

The objective of this study was to evaluate the association of LTG plasma concentrations with clinical efficacy and toxicity in patients with epilepsy. In addition, the dose–concentration relationship of LTG was assessed. Consequently, an optimal reference range for LTG was proposed, with the aim of improving the efficacy and safety of treatment.

## MATERIALS AND METHODS

### Study Design and Population

This retrospective study was conducted at the University Medical Center Utrecht, the Netherlands. Inpatients and outpatients with epilepsy, for whom a LTG concentration was measured between January 2013 and February 2022, were included. Patients were excluded if LTG was used for the treatment of indications other than seizures (e.g., bipolar disorder). Patients with undetectable LTG plasma concentrations were also excluded. If LTG plasma concentrations were measured multiple times in the same patient on the same day, trough concentrations were used, and the other concentrations were excluded. This study was approved by the local Medical Research Ethics Committee (research proposal 22/086), and all included patients consented to the use of their data for research purposes.

LTG plasma concentrations were measured at the laboratory of the Department of Clinical Pharmacy of the University Medical Center Utrecht using a standard liquid chromatography–tandem mass spectrometry (LC-MS/MS) method, which was validated according to the Guideline on Bioanalytical Method Validation of the European Medicines Agency.^13^ In general, LTG TDM was performed upon indication and not routinely, in accordance with treatment guidelines.^14^

### Data Collection

The laboratory information system was used for retrieving all LTG plasma concentration measurements available within the retrospective time frame, including data on the date and time of blood sampling. Each patient's digital health records were reviewed to collect the following patient-specific and medication-related characteristics: age, sex, pregnancy, weight, department where the patient was admitted, reason for TDM, LTG use (indication, dose, last administration of LTG before blood sampling), and concomitant antiepileptic drugs. The following drugs were classified as LTG-metabolizing enzyme inducers: carbamazepine, phenobarbital, phenytoin, oxcarbazepine, and estrogen-containing/combined oral contraceptives. VPA was considered as an LTG-metabolizing enzyme inhibitor.^5,9^

For each LTG plasma concentration, patient data were collected over a 2-year time frame: 1 year before blood sampling until 1 year afterward. Data closest to the moment of blood sampling were used for the analysis.

### Efficacy and Toxicity of LTG

The association of LTG plasma concentration with the efficacy and toxicity of treatment was assessed. Efficacy was defined as SF for at least 6 months around the time of blood sampling. Each patient was included once, and the first recorded LTG plasma concentration was used. When multiple LTG measurements from the same patient were available within 1 year after the first included plasma concentration, the average LTG plasma concentration over this period was used for further analysis. The average plasma concentration was used to correct for any intraindividual fluctuations in LTG plasma concentrations, whether this was due to different blood sampling times or not. Plasma concentrations measured within the 1-year period were included regardless of dosage changes. Individual LTG concentrations measured outside the 1-year period were excluded from the analysis.

Toxicity was defined as any potential LTG-related adverse drug effect documented in each patient's health record or when the reason for measuring the LTG concentration was suspected toxicity. Toxicity symptoms were categorized into 4 major groups, according to the commonly reported adverse effects of LTG: (1) neurological effects, including headache, dizziness, drowsiness, aphasia, poor concentration, poor memory, psychomotor slowing, cognitive slowing, confusion/disorientation, and word-finding difficulty; (2) gastrointestinal effects, including nausea, vomiting, diarrhea, and loss of appetite; (3) dermatological effects including rash; and (4) other adverse effects, including visual effects (diplopia, nystagmus), motor effects (ataxia, imbalance, tremor), and psychiatric effects (changes in behavior or mood).^6–8^

Toxicity symptoms, if any, must have been present on the day of blood sampling. Otherwise, data closest to the time of blood sampling and documented within 2 weeks afterward were used. When multiple LTG plasma concentrations from the same patient were available within 6 months, the average of consecutively measured concentrations for which toxicity was reported was used in the analysis. When no toxicity was reported, the average concentration was used as well. By using consecutively measured concentrations when toxicity was present, the possibility of missing specific concentrations for toxicity requests was minimized. When the presence or category of toxicity varied between multiple LTG concentrations for a patient, each combination of an (average) LTG concentration and toxicity data was included in the analysis.

The dose–concentration relationship was evaluated by correlating all LTG plasma concentrations with the corresponding cumulative daily doses of LTG.

### Data Analysis

Descriptive statistics were used to summarize patient-related and medication-related characteristics, using the data of the first included LTG concentration of each patient. Data are presented as medians with ranges or as means. Categorical variables are expressed as numbers with percentages, n (%).

Univariate logistic analysis was used to explore the influence of several covariates on achieving SF ≥ 6 months, the occurrence of toxicity, and on the LTG plasma concentration, by which potential confounders and/or effect modifiers were identified. Based on biological plausibility and the available literature, the following covariates were selected: age, sex, pregnancy, seizure type, concomitant use of interacting drugs, and number of antiepileptic drugs. Age, sex, and other variables with *P* ≤ 0.20 were then included in the multivariate model. Multivariate logistic regression analysis was performed to examine the correlation of the LTG plasma concentration with the odds of achieving SF ≥ 6 months and the occurrence of toxicity. Linear regression analysis was used to study the relationship between the daily LTG dose and LTG plasma concentration. All data were analyzed using SPSS Statistics version 28.0 (IBM, Armonk, NY). A *P*-value < 0.05 was considered statistically significant.

## RESULTS

### Patient Characteristics and LTG Plasma Concentrations

Overall, 549 LTG plasma concentrations from 259 patients were included in this study, with an average of 2 concentrations per patient (range, 1–20 per patient). Characteristics of the patient population and antiepileptic treatments are presented in Table 1. The median age of the patients was 33 years (range, 1–84 years). Most patients (38%) were receiving LTG monotherapy when the LTG concentration was first measured. The median LTG daily dose was 250 mg (range, 20–800 mg). Patients were using LTG once (5%), twice (92%), 3 (2%), or 4 (1%) times daily.

**TABLE 1.** Patient Characteristics and Antiepileptic Treatment

	Total Patients (n = 259)
Age (yrs, %)	33 (1–84)
Younger than 18	43 (16.6)
18–60	173 (66.8)
Older than 60	43 (16.6)
Body weight (kg, %)	68 (12–155)
<50	37 (17.7)
50–75	90 (43.1)
>75	82 (39.2)
Sex	
Men	123 (47.5)
Women	136 (52.5)
Pregnant	10 (7.4)
Seizure type (%)	
Focal onset	134 (51.7)
Generalized	84 (32.4)
Unknown	41 (15.8)
Antiepileptic treatment (%)	
LTG dose (mg/d)	250 (20–800)
No VPA and/or enzyme inducers*	155 (59.8)
LTG + VPA	44 (17.0)
LTG + enzyme inducers*	51 (19.7)
LTG + VPA and enzyme inducers*	9 (3.5)
Number of antiepileptic drugs (%)	
1 (LTG monotherapy)	98 (37.8)
2	84 (32.4)
3	45 (17.4)
4	25 (9.7)
5	7 (2.7)
Hospital department (%)	
Outpatients	160 (61.8)
Neurology	48 (18.5)
Emergency care	29 (11.2)
Intensive care	3 (1.2)
Other†	19 (7.3)

* Enzyme inducers: carbamazepine, phenobarbital, phenytoin, oxcarbazepine, and combined oral contraceptives.

† Other departments: cardiology, cerebrovascular diseases, hematology, geriatrics, internal medicine, obstetrics, surgery, urology.

In patients using LTG once daily, 15% of blood samples were drawn at 0–4 hours after administration, 22% at 4–12 hours, 22% at 12–20 hours, 22% at 20–25 hours, and 19% at unknown time points. In patients using LTG twice daily, 9% of blood samples were drawn at 0–4 hours after administration, 27% at 4–10 hours, 51% at 10–19 hours, and 12% at unknown timepoints (see **Supplemental Digital Content 1**, http://links.lww.com/TDM/A729, which illustrates LTG plasma concentrations at different time points after administration following a twice-daily dosing regimen).

All LTG plasma concentrations and the reasons for TDM are presented in Table 2. Most of the plasma concentrations (62%) were assessed in outpatients. The median LTG concentration was 3.6 mg/L (range, 0–25 mg/L), with 68% of the concentrations in the lowest range of 0.1–4.9 mg/L. Roughly half (53%) of the LTG concentration assessments were trough concentrations. The most common reasons for TDM were suspected inefficacy (39%) and monitoring before/during/after pregnancy (21%).

**TABLE 2.** LTG Plasma Concentrations and Reasons for TDM

	Total LTG Concentrations (n = 549)
LTG concentration (mg/L, %)	
0.1–4.9	374 (68.1)
5.0–9.9	122 (22.2)
10.0–14.9	42 (7.7)
15.0–25.0	11 (2.0)
Reason for TDM (%)	
Inefficacy	212 (38.6)
Toxicity	55 (10.0)
Seizures and toxicity	14 (2.6)
Pregnancy (before, during, and postpartum)	113 (20.6)
Routine check	52 (9.5)
Interaction	39 (7.1)
LTG dose titration	47 (8.6)
Nonadherence	14 (2.6)
Other (anesthesia, intake problems)	3 (0.5)

**FIGURE 1.**
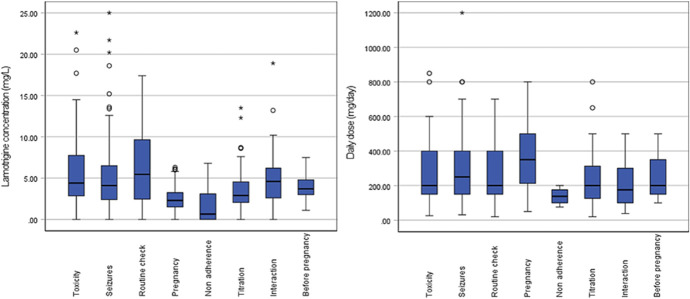
Boxplots of the LTG concentration (left) and daily dose (right) per reason for TDM.

Median LTG concentrations categorized by reason for TDM are presented in boxplots (Fig. 1). Notably, there is an overlap between the LTG concentrations measured because of seizures and toxicity. The median LTG plasma concentration was < 5 mg/L, and the daily LTG dose was higher in women who were pregnant than in individuals with other reasons for TDM.

### LTG Plasma Concentration–Efficacy Relationship

**FIGURE 2.**
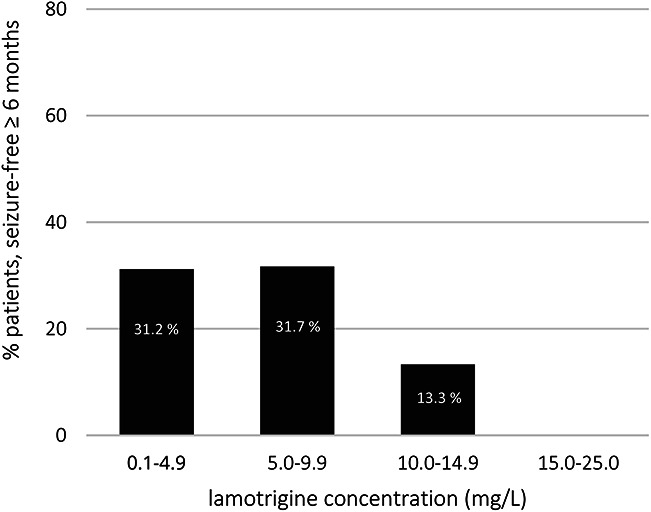
Percentages of patients who were seizure-free ≥ 6 months by range of LTG concentration.

The efficacy of LTG treatment was not assessable in 5 of the 259 included patients since the follow-up period was shorter than 6 months. Of the remaining 254 patients, 75 (30%) achieved SF ≥ 6 months. The mean LTG concentration in patients without seizures was 4.0 mg/L versus 4.7 mg/L in patients with seizures within 6 months (*P* = 0.15). In patients with LTG concentrations 5.0–9.9 mg/L, 31.7% achieved SF, while in those with concentrations ≥ 10 mg/L, 13.3% achieved SF (Fig. 2). The LTG concentration was not significantly associated with SF ≥ 6 months (*P* = 0.15; see **Supplemental Digital Content 2**, http://links.lww.com/TDM/A730, which shows the results of univariate analyses of the association between covariates and efficacy), which was also true after adjusting for age, sex, and the number of antiepileptic drugs (adjusted odds ratio [OR] = 0.94; 95% confidence interval [CI], 0.85–1.04; *P* = 0.21; see **Supplemental Digital Content 3**, http://links.lww.com/TDM/A731, which shows the multivariate model of the association between covariates and efficacy).

The proportion of patients without seizures was higher when using LTG monotherapy as an antiseizure treatment (42%), compared with that when using dual (32%) or triple (12%) therapy. The results of univariate analysis showed that the number of antiepileptic drugs was negatively associated with efficacy (see **Supplemental Digital Content 2**, http://links.lww.com/TDM/A730). After adjusting for age and sex in the multivariate analysis, the odds of SF ≥6 months were significantly lower with treatment with at least 3 antiepileptic drugs than with LTG monotherapy (OR = 0.21; 95% CI, 0.09–0.47; see **Supplemental Digital Content 3**, http://links.lww.com/TDM/A731).

Age was positively associated with SF ≥ 6 months (OR = 1.02; 95% CI, 1.00–1.03). Only 7% of children (younger than 18 years) were seizure-free, while higher proportions of patients aged 18–60 and older than 60 years were seizure-free (32% and 45%, respectively).

### LTG Plasma Concentration–Toxicity Relationship

A total of 299 LTG plasma concentrations were included in the analysis. Toxicity symptoms associated with LTG were reported for 35% of the LTG concentrations (n = 104/299). Cognitive symptoms, including dizziness, confusion, fatigue, slowed thinking and speech, impaired memory, and sleep disorders, accounted for the highest percentage (23%) of reported adverse effects. Motor symptoms were reported for 15% of the LTG concentrations, which included tremor, loss of coordination, unsteadiness, and difficulty walking. Gastrointestinal symptoms, including nausea, vomiting, and diarrhea, were reported for 5.7% of the concentrations and skin rash for 3%. Other reported adverse effects (6.4%) included diplopia, nystagmus, vision problems, dry mouth, muscle ache, and palpitations.

**FIGURE 3.**
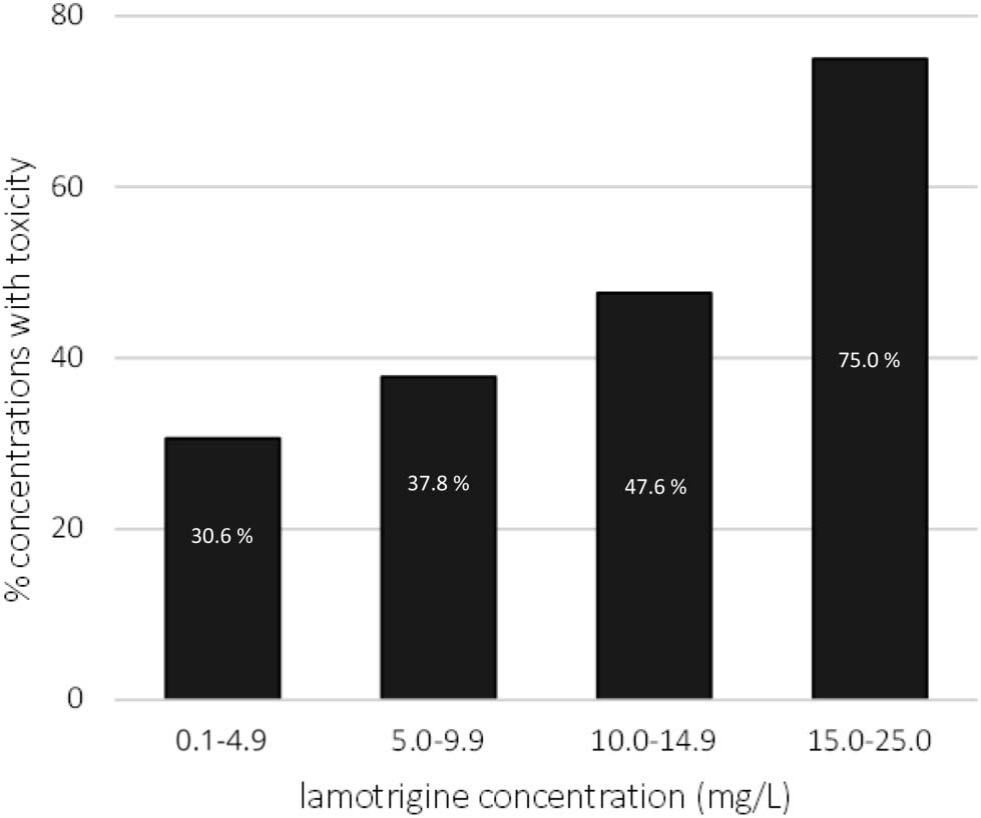
Percentages of LTG concentrations in which toxicity was reported by range of LTG concentration.

The mean LTG concentration for which toxicity was reported was 5.7 mg/L versus 4.2 mg/L when no toxicity was reported (*P* = 0.001). The incidence of toxicity increased with elevating LTG concentrations (Fig. 3). Univariate analysis showed that LTG plasma concentration was positively associated with toxicity; every increase of 1 mg/mL in LTG concentration was associated with an 11% increase in the odds of toxicity (OR = 1.11; 95% CI, 1.04–1.18); see **Supplemental Digital Content 4**, http://links.lww.com/TDM/A732, which illustrates the results of univariate analyses of the association between covariates and toxicity). This correlation was statistically significant after adjusting for age, sex, and the number of antiepileptic drugs (OR = 1.11; 95% CI, 1.04–1.19). Moreover, the risk of toxicity was significantly higher in patients treated with at least 3 antiepileptic drugs than in patients treated with LTG alone (OR = 2.99; 95% CI, 1.58–5.64). For the full multivariate model of the association between covariates and toxicity, see **Supplemental Digital Content 5** (http://links.lww.com/TDM/A733).

### Dose–Concentration Relationship of LTG

The daily LTG dose had a significant linear correlation with the LTG plasma concentration (*P* < 0.001). Pregnancy was identified as a relevant effect modifier (*P* = 0.01), and thus, a stratified analysis of this variable was conducted in women. The B-coefficient indicated that a daily dose increase of 100 mg of LTG resulted in an average increase in the LTG concentration of 1.2 mg/L in women who were not pregnant and 0.5 mg/L in women who were pregnant (see **Supplemental Digital Content 6**, http://links.lww.com/TDM/A734, which illustrates the multivariate linear models of the association of LTG dose and other covariates with the plasma concentration). In men, the B-coefficient was equal to that in women who were not pregnant.

In women who were not pregnant, concomitant use of VPA or enzyme inducers was also significantly associated with the LTG concentration when compared with no concomitant use of interacting drugs. This variable could not be evaluated in women who were pregnant, since they were all using LTG without VPA and/or enzyme inducers. Weight was available for 211 out of the 259 patients and did not contribute to the adjusted R^2^ when it was added to the multivariate models.

## DISCUSSION

In this retrospective study, an association between the LTG plasma concentration and toxicity was demonstrated. A significant, independent, and positive association of the toxicity of LTG was found after adjusting for age, sex, and the number of antiepileptic drugs.

No association between the LTG plasma concentration and efficacy was found in this study. However, with inefficacy as the most common reason for TDM (Table 2), the potential for finding a possible association with plasma concentration may have been attenuated. This is due to potential selection bias for patients who were not seizure-free. In addition, according to national treatment guidelines,^14^ LTG plasma concentrations were assessed based on indication. Thus, patients who were seizure-free were less likely to have their plasma concentration assessed. The literature is inconsistent about the therapeutic concentration range of LTG. Froscher et al found at least 50% seizure reduction at a median LTG concentration of 3.6 mg/L (range, 1.3–7.1 mg/L). By contrast, Schapel et al suggested a therapeutic window of 8–16 mg/L using the median LTG concentrations, where 50% seizure reduction and the occurrence of toxicity were used as the lower and upper limits, respectively.^11,12^

As reported by Hirsch et al,^6^ SF was mostly observed in patients with LTG concentrations below 10 mg/L (Fig. 2). In patients with refractory seizures, higher doses and concentrations may be needed. This may explain the variety of efficacious concentrations and the lack of correlation. Hirsch et al^6^ suggested that higher LTG concentrations could lead to additional efficacy in patients who were refractory: Approximately 30% of patients were seizure-free for 6 months and had an LTG concentration of 15–20 mg/L.

In this study, undetectable plasma concentrations (n = 21) were excluded, thus potentially overlooking TDM requests due to poor adherence and/or poor response. However, poor patient adherence was only suspected for 4 plasma concentrations. Other reasons for TDM included pregnancy (n = 10), LTG in titration (n = 3), and interactions (n = 4). Excluding undetectable levels was anticipated to have little impact on the present findings.

Age was found to be a relevant predictor for achieving SF. SF was achieved in a relatively small proportion of children, compared with adults and older patients. This can be attributed to the unconventional use of LTG for treating children with epilepsy. According to national treatment guidelines,^14^ treatment with LTG is only initiated in children with refractory epilepsy.

In accordance with previous studies,^6,8^ an association was found between the LTG plasma concentration and toxicity. The incidence of toxicity increased 2-fold when the LTG concentration was above 15 mg/L compared with that at a concentration of 5.0–9.9 mg/L (75% versus 38%; Fig. 3). This finding is comparable with those from other studies, which reported a 3-fold increase in the incidence of toxicity above an LTG concentration of 13 mg/L.^8,12^

Age was positively associated with LTG toxicity. Possible explanations for a greater susceptibility to toxicity in older patients are polytherapy and fragility. The number of antiepileptic drugs was also positively associated with LTG toxicity. On the contrary, Hirsch et al^6^ found similar incidences of toxicity between patients using varying numbers of drugs. Similar to our study, the range of LTG concentrations that Hirsch et al assessed was 0–25 mg/L, with most <10 mg/L.

However, variability in the findings may be due to different definitions of toxicity. Hirsch et al defined toxicity as adverse effects that led to dosage change or discontinuation of LTG, while in the present study, all potential LTG-related adverse effects were included.

In line with previous findings,^15^ a linear correlation between the daily LTG dose and LTG plasma concentration was demonstrated. An increase in the LTG dose resulted in a greater average increase in the LTG concentration in women who were not pregnant than in those who were. This is consistent with the finding that LTG clearance is increased up to 300% in women who are pregnant, which is caused by induction of LTG metabolism due to physiological changes.^16^ In line with previous findings,^6^ the LTG concentration at a given dose was increased with the concomitant use of VPA, while concomitant use of enzyme-inducing drugs decreased the LTG concentration.

The strengths of this study include the heterogenous study population of patients with epilepsy who were under treatment at an academic hospital, which reflects the real world and contributes to the generalizability of the data. This study has several limitations. First, since TDM is not performed routinely for LTG, except during pregnancy,^14^ selection bias would have been introduced; patients without an indication for assessing their LTG plasma concentration were less likely to be included in the study (e.g., patients who were seizure-free). Second, nearly half of the measurements were not trough concentrations. Nevertheless, little impact on the findings was anticipated; although little is known about the magnitude of the intraindividual variation in LTG concentrations, the relatively long half-life of LTG (33 hours) minimizes variations between peak and trough concentrations (also illustrated in **Supplemental Digital Content 1**, http://links.lww.com/TDM/A729). Furthermore, this might contribute to the generalizability of the findings since measuring trough concentrations is not always achievable in practice. Finally, the study's retrospective design may have contributed to potential underreporting of both toxicity and efficacy endpoints.

This study contributes to evidence for refining the LTG therapeutic reference range. The discrepancy in previously suggested reference ranges may be due to differences in assessed outcomes; while some studies considered either efficacy or toxicity, some considered both. The current reference range found in national guidelines is 2.5–15 mg/L. Based on this study, a narrower LTG reference range of 2.5–10 mg/L may be considered to decrease the risk of toxicity, while having a similar effect on seizures in most patients.

TDM of LTG is warranted for several reasons. Considering the high interpatient variability,^17^ measuring individual LTG concentrations may be beneficial in patients initiating or discontinuing interacting drugs, pregnancy, or in cases when LTG-related toxicity is suspected.

## CONCLUSIONS

An association between the LTG plasma concentration and toxicity was demonstrated, with older patients using multiple antiepileptic drugs being at the highest risk for adverse effects. No association between the LTG plasma concentration and the efficacy of treatment was found. The LTG dose showed a significant and linear correlation with the LTG plasma concentration, which was lower in women who were pregnant and patients who used concomitant enzyme inducers at a given LTG dose. The linear correlation indicates that LTG dose adjustments can be easily guided by plasma concentrations. Based on the present findings, an LTG reference range of 2.5–10 mg/L may be considered to decrease the risk of toxicity while having a similar effect on seizures in most patients. TDM may be useful when LTG-related toxicity is suspected and in cases of anticipated pharmacokinetic changes.

## Supplementary Material

SUPPLEMENTARY MATERIAL
