# Supplementary material for: Association of Lamotrigine Plasma Concentrations With Efficacy and Toxicity in Patients With Epilepsy: A Retrospective Study
Source: Ther Drug Monit. 2024 Jun 28;46(5):642–8. doi: 10.1097/FTD.0000000000001205 (PMC11389884; doi:10.1097/FTD.0000000000001205)
Supplement: SUPPLEMENTARY MATERIAL [file tdm-46-642-s001.docx]

**Supplemental Digital Content 1.** Lamotrigine plasma concentrations (corrected for daily dose, per 100 mg) at different timepoints after administration following a twice-daily dosing regimen.
