# Supplementary material for: Association of Lamotrigine Plasma Concentrations With Efficacy and Toxicity in Patients With Epilepsy: A Retrospective Study
Source: Ther Drug Monit. 2024 Jun 28;46(5):642–8. doi: 10.1097/FTD.0000000000001205 (PMC11389884; doi:10.1097/FTD.0000000000001205)
Supplement: SUPPLEMENTARY MATERIAL [file tdm-46-642-s002.docx]

**Supplemental Digital Content 2.** Univariate analyses of the association between the lamotrigine plasma concentration, age, gender, seizure type, and the number of antiepileptic drugs with seizure freedom ≥ 6 months.

| **All patients (n = 254)** | | | | |
| --- | --- | --- | --- | --- |
|  | **Total (n)** | **SF ≥ 6 months, n (%)** | **OR** | **95% CI** |
| LTG concentration (mg/L) | 254 | 75 (29.5%) | 0.94 | 0.86-1.02 |
| 0.0-4.9 | 170 | 53 (31.2%) | Ref |  |
| 5.0-9.9 | 63 | 20 (31.7%) | 1.03 | 0.55-1.91 |
| 10.0-14.9 | 15 | 2 (13.3%) | 0.34 | 0.07-1.56 |
| 15.0-25.0 | 5 | 0 (0.0%) | 0.00 |  |
| Age (yr) | 254 | 75 (29.5%) | 1.02 | 1.01-1.04 |
| < 18 | 43 | 3 (6.98%) | Ref |  |
| 18-60 | 171 | 54 (31.6%) | 6.15 | 1.82-20.78 |
| > 60 | 40 | 18 (45.0%) | 10.91 | 2.89-41.18 |
| Gender (male) | 122 | 32 (26.2%) | Ref |  |
| Gender (female) | 132 | 43 (32.6%) | 1.36 | 0.79-2.34 |
| Seizure type | 213 | 63 (29.4%) |  |  |
| Focal | 130 | 39 (30.0%) | Ref |  |
| Generalized | 83 | 22 (26.5%) | 0.84 | 0.46-1.56 |
| Number of antiepileptic drugs |  |  |  |  |
| 1 | 95 | 40 (42.1%) | Ref |  |
| 2 | 82 | 26 (31.7%) | 0.64 | 0.34-1.19 |
| ≥3 | 77 | 9 (11.7%) | 0.18 | 0.08-0.41 |

LTG concentration and age were analyzed as continuous and categorical variables. OR: odds ratio. CI: confidence interval for odds ratio. Ref: reference. LTG: lamotrigine. SF: seizure freedom.
