# Supplementary material for: Association of Lamotrigine Plasma Concentrations With Efficacy and Toxicity in Patients With Epilepsy: A Retrospective Study
Source: Ther Drug Monit. 2024 Jun 28;46(5):642–8. doi: 10.1097/FTD.0000000000001205 (PMC11389884; doi:10.1097/FTD.0000000000001205)
Supplement: SUPPLEMENTARY MATERIAL [file tdm-46-642-s003.docx]

**Supplemental Digital Content 3.** Multivariate model of the association between lamotrigine plasma concentration and seizure freedom ≥ 6 months.

|  | **All patients (n = 254)** | |
| --- | --- | --- |
|  | **OR** | **95% CI** |
| LTG concentration (mg/L) | 0.94 | 0.85-1.04 |
| Age (y) | 1.02 | 1.00-1.03 |
| Gender (female) | 1.19 | 0.67-2.15 |
| Number of antiepileptic drugs |  |  |
| 1 | Ref |  |
| 2 | 0.71 | 0.37-1.36 |
| ≥3 | 0.21 | 0.09-0.47 |

LTG: lamotrigine. OR: odds ratio. CI: confidence interval for odds ratio.
