# Supplementary material for: Association of Lamotrigine Plasma Concentrations With Efficacy and Toxicity in Patients With Epilepsy: A Retrospective Study
Source: Ther Drug Monit. 2024 Jun 28;46(5):642–8. doi: 10.1097/FTD.0000000000001205 (PMC11389884; doi:10.1097/FTD.0000000000001205)
Supplement: SUPPLEMENTARY MATERIAL [file tdm-46-642-s004.docx]

**Supplemental Digital Content 4.** Univariate analyses of the association between the lamotrigine plasma concentration, age, gender, pregnancy, and number of the antiepileptic drugs with toxicity.

| **LTG concentrations (n = 299)** | | | | |
| --- | --- | --- | --- | --- |
|  | **Total (n)** | **Toxicity, n (%)** | **OR** | **95% CI** |
| LTG concentration (mg/L) | 299 | 104 (34.8%) | 1.11 | 1.04-1.18 |
| 0.0-4.9 | 196 | 60 (30.6%) | Ref |  |
| 5.0-9.9 | 74 | 28 (37.8%) | 1.38 | 0.79-2.41 |
| 10.0-14.9 | 21 | 10 (47.6%) | 2.06 | 0.83-5.11 |
| 15.0-25.0 | 8 | 6 (75.0%) | 6.80 | 1.33-34.7 |
| Age (yr) | 299 | 104 (34.8%) | 1.01 | 1.00-1.02 |
| < 18 | 45 | 15 (33.3%) | Ref |  |
| 18-60 | 201 | 67 (33.3%) | 1.00 | 0.50-1.99 |
| > 60 | 53 | 22 (41.5%) | 1.42 | 0.62-3.24 |
| Gender (male) | 141 | 51 (36.2%) | Ref |  |
| Gender (female) | 158 | 53 (33.5%) | 0.89 | 0.55-1.44 |
| Number of antiepileptic drugs |  |  |  |  |
| 1 | 109 | 26 (23.9%) | Ref |  |
| 2 | 99 | 36 (36.4%) | 1.82 | 1.00-3.33 |
| ≥3 | 91 | 42 (46.2%) | 2.74 | 1.50-5.00 |

LTG concentration and age were analyzed as continuous and categorical variables. OR: odds ratio. CI: confidence interval for odds ratio. Ref: reference. LTG: lamotrigine.
