# Supplementary material for: Association of Lamotrigine Plasma Concentrations With Efficacy and Toxicity in Patients With Epilepsy: A Retrospective Study
Source: Ther Drug Monit. 2024 Jun 28;46(5):642–8. doi: 10.1097/FTD.0000000000001205 (PMC11389884; doi:10.1097/FTD.0000000000001205)
Supplement: SUPPLEMENTARY MATERIAL [file tdm-46-642-s005.docx]

**Supplemental Digital Content 5.** Multivariate model of the association between the lamotrigine plasma concentration and toxicity.

|  | **LTG concentrations (n = 299)** | |
| --- | --- | --- |
|  | **OR** | **95% CI** |
| LTG concentration (mg/L) | 1.11 | 1.04-1.19 |
| Age (y) | 1.02 | 1.00-1.03 |
| Gender (female) | 0.99 | 0.60-1.65 |
| Number of antiepileptic drugs |  |  |
| 1 | Ref |  |
| 2 | 1.56 | 0.83-2.92 |
| ≥3 | 2.99 | 1.58-5.64 |

LTG: lamotrigine. OR: odds ratio. CI: confidence interval for odds ratio.
