# Supplementary material for: Association of Lamotrigine Plasma Concentrations With Efficacy and Toxicity in Patients With Epilepsy: A Retrospective Study
Source: Ther Drug Monit. 2024 Jun 28;46(5):642–8. doi: 10.1097/FTD.0000000000001205 (PMC11389884; doi:10.1097/FTD.0000000000001205)
Supplement: SUPPLEMENTARY MATERIAL [file tdm-46-642-s006.docx]

**Supplemental Digital Content 6.** Multivariate linear models of the association of lamotrigine dose, age, gender, enzyme-inhibitors and/or enzyme-inducers with the lamotrigine plasma concentration.

| **Variable** | **B-coefficient** | **95% CI** |
| --- | --- | --- |
| **All concentrations (n = 549)** | | |
| Constant | 1.72 | 0.85-2.60 |
| Dose (mg/day) | 0.009 | 0.007-0.01 |
| Age (y) | 0.003 | - 0.01-0.02 |
| Gender (female) | - 0.57 | - 0.01-0.02 |
| No VPA and/or inducers* | Ref |  |
| LTG + VPA | 4.92 | 4.12-5.71 |
| LTG + inducers* | - 1.51 | - 2.31; -0.71 |
| LTG + VPA and inducers* | 1.33 | - 0.38-3.03 |
| **Non-pregnant women (n = 232)** | | |
| Constant | 1.73 | 0.50-2.95 |
| Dose (mg/day) | 0.012 | 0.010-0.014 |
| Age (y) | - 0.01 | - 0.03-0.02 |
| No VPA and/or enzyme-inducers* | Ref |  |
| LTG + VPA | 4.37 | 3.05-5.68 |
| LTG + enzyme-inducers* | - 2.27 | -3.56; -0.97 |
| LTG + VPA and enzyme-inducers* | 0.27 | -2.56-3.09 |
| **Pregnant women (n = 94)** | | |
| Constant | - 2.35 | -4.00; -0.70 |
| Dose (mg/day) | 0.005 | 0.004-0.006 |
| Age (y) | 0.08 | 0.03-0.13 |

CI: confidence interval for the B-coefficient. LTG: lamotrigine. VPA: valproic acid. Ref: reference. *Enzyme-inducers: carbamazepine, phenobarbital, phenytoin, oxcarbazepine, and combined oral contraceptives.
